# Community experience and awareness regarding foreign body aspiration in Asir region, Kingdom of Saudi Arabia

**DOI:** 10.1097/MD.0000000000038869

**Published:** 2024-08-02

**Authors:** Ayoub Ali Alshaikh, Abdullah Alhelali, Abdulrahim Ali Hassan, Mohammed Yahya Althwabi Asiri, Lujain Khaled T. Bukhari, Abduaelah Ali H. Hassan, Abeer Ali H. Hassan, Reem Saud H. Alqahtani, Raghad Yahya AlQahtani, Ramy Mohamed Ghazy

**Affiliations:** a Family & Community Medicine Department, King Khalid University, College of Medicine Abha, Abha, Saudi Arabia; b Abha Children Hospital, Aseer Central Hospital, Head of Airway/Laryngology Unit – Aseer, Central Hospital, Abha, Saudi Arabia; c Otorhinolaryngology, Head and Neck Surgery, Armed Forced Hospital Southern Region, Khamis Mushait, Saudi Arabia; d Family Medicine, Abha, Saudi Arabia; e Medical Intern, King Khalid University, Abha, Saudi Arabia; f King Khalid University, Abha, Saudi Arabia; g Tropical Health Department, High Institute of Public Health, Alexandria University, Alexandria, Egypt.

**Keywords:** community awareness, foreign body aspiration, foreign body inhalation, morbidity, mortality, Saudi Arabia

## Abstract

Foreign body aspiration (FBA) is a serious preventable pediatric health problem and one of the main causes of accidental death in children. Although unusual in adults, it is often overlooked as a cause of airway obstruction with serious consequences. This study assessed awareness and previous FBA experiences in the Asir community, Kingdom of Saudi Arabia. Using validated questionnaire, an annonymous online survey was conducted among 870 people aged 18 years and above. The questionnaire was used to collect data about the personal and sociodemographic characteristics of the respondents, as well as their experiences with FBA, and participants’ knowledge and perceived seriousness of FBA. The level of knowledge was deemed good if the score ranged between 60% and 100%, and bad if the score fell below 60%The level of knowledge was deemed good if the score ranged between 60% and 100%, and bad if the score fell below 60%. The majority of the participants (79.7%) were females, 48.1% were aged 18 to 30 years, 72.9% had university degree, 30.6% were students, 26.9% worked in the educational sector, 43.6% reported monthly income of <5000 Saudi Riyals and 19.8% of them identified themselves as healthcarepractitioners. Although the community experience with FBA was considerably high (70.6%) among the study participants, their awareness levels about FBA were deficient. Only 24.7% of the respondents had good knowledge of FBA. Older age, being a health practitioner, and perceiving FBA as a serious incident were significantly associated with good knowledge (*P* < .001). The findings of this study indicate an urgent need to raise community awareness of FBA. To reduce FBA morbidity and mortality, health education efforts in community and healthcare settings are required to educate people about the seriousness and importance of early diagnosis and management of the condition.

## 1. Introduction

Foreign body aspiration (FBA) occurs when solid objects block the airway at the level of the glottis, larynx, trachea, or bronchi.^[1]^ Serious immediate and delayed complications are associated with FBA, depending on the level of obstruction and duration of retention. These complications range from recurrent pneumonia to respiratory or cardiac arrest.^[2]^ FBA is one of the major preventable causes of morbidity and mortality in children, ranging from 10% to 20% worldwide.^[3]^ FBA is considered the 5th leading cause of accidental death in children younger than 3 years and the main cause of death in infants younger than 1 year.^[4,5]^ The overall death rate associated with FBA is 5% to 7%.^[6]^

The incidence of FBA is higher in preschool children than in adults and older children. The highest frequency of FBA occurs between one and 3 years of age.^[4,7]^ Several factors contribute to the increased risk of choking in young children. These include inadequate supervision by adults. Young children tend to explore their environment by putting objects in their mouths, which increases the risk of choking while they are talking, playing, or eating. Other factors may include an underdeveloped swallowing mechanism, the absence of molar teeth, and immature neuromuscular mechanisms of airway protection.^[8–10]^

The nature of aspirated foreign bodies varies among different populations according to culture, dietary habits, and lifestyle.^[11–13]^ Globally, organic products including peanuts, beans, seeds, and nuts are the predominant aspirated substances in children.^[10,14]^ “Inorganic substances make up only 20.4% of foreign body aspiration cases. The most common non-food foreign bodies include metal objects (toys, springs, hair clips, and paper clip earrings), plastic objects (ballpoint pen tips, pencil caps, and whistles), magnets, batteries, and latex balloons.”^[15,16]^ Organic foreign bodies tend to cause more inflammation in the local mucosa and form granulation tissue within a few hours of aspiration.^[14]^

Timely diagnosis of FBA is a challenge for physicians. Delayed diagnosis or misdiagnosis is common due to the lack of specific signs or symptoms. The signs and symptoms of FBA can mimic other diseases such as asthma, pneumonia, and croup.^[5]^ Moreover, approximately 15% to 20% of patients may not have any signs or symptoms.^[17]^

Higher rates of serious complications are associated with delayed presentation, diagnosis, or management.^[18]^ Foreign bodies can remain undetected for a long time and cause serious complications if not diagnosed promptly. These complications may include respiratory distress, asphyxia, pneumonia, atelectasis, bronchiectasis, hypoxic brain injury, and even death. So that it is crucial to diagnose, and address retained foreign bodies on time to prevent these complications.^[3,5,19,20]^

Preventing FBA is more important than its treatment.^[21]^ Caregivers and those in direct contact with young children are the first to recognize and deal with a child who is choking. For adults, it is crucial to be aware of this health problem and its complication so that they would seek treatment as soon as possible. This emphasizes the importance of assessing and raising community awareness about the management and consequences of FBA including its signs and symptoms and the knowledge of preventive measures. Increasing community awareness is essential for the early and proper diagnosis and management of choking incidents. Some countries have implemented health education campaigns as a public health measure, which have successfully reduced the rates of choking incidents and associated mortality.^[22–24]^ This study aimed to assess the experience and the level of community awareness of FBA in the Asir region of the Kingdom of Saudi Arabia (KSA). The results of this study will contribute to the existing evidence and assist in developing suitable educational interventions.

## 2. Methodology

### 2.1. Study design and setting

A descriptive anonymous online cross-sectional study was conducted to assess community experience and awareness of FBA among residents of the Asir region, KSA. The study was conducted over 8 months from May 1, 2023, to December 31, 2023.

### 2.2. Study population and sample size

Using Epi-Info software, we estimated that a minimum of 500 participants would be needed for the study. This estimation was based on a 5% margin of error, 95% confidence interval, and a 30% nonresponse rate, assuming that 50% of the population has a fair level of knowledge and a 30% response rate. The study included the population over 18 years who lived in the Asir region during the study period. Convenient and snowballing non-probability sampling techniques were used to enroll Asir residents in the study.

### 2.3. Data collection tool

Participants who agreed to take part in the study were asked to complete an online questionnaire after being provided with a clear explanation of the study’s objective. The questionnaire was uploaded to Google formand the link was distributed through commonly used social media platforms (Instgram, Messenger, WhatsApp, and facebook). The questionnaire consisted of 3 sections. The first section collected personal and sociodemographic data, including age, gender, education, occupation, and income. The second section focused on the participants’ experiences with FBA. They were asked if they had ever aspirated a foreign body or knew someone who had, the age at which the incident occurred, the type of foreign body (organic [food/plants], inorganic [minerals], electromagnetic [batteries], or other), and how the foreign body was managed. Finally, the third section covered participants’ knowledge and perceived seriousness of FBA. Knowledge was assessed through a series of questions regarding:

- Practices and recommendations for prevention of FBA in children (10 questions): participants answered 10 yes/no items regarding correct preventive practice. By coding yes = 1 and no = 0, a maximum total preventive knowledge score of 10 was calculated.
- Priority life-saving recommendations for the management of FBA in infants and adults (3 questions): participants were asked about the Heimlich maneuver (an illustrative photo was used), trying to get it out by hand encouraging the aspirated person to cough, turning the baby down (if the infant) and back blows and chest thrust (if adult). Three questions were used to assess knowledge about the management of FBA. Correct responses are coded as 1 and incorrect ones are coded as 0.
- Common symptoms of FBA (8 questions): participants were asked to answer 8 right/wrong items about symptoms of FBA. Each right symptom mentioned was coded as 1 where a maximum score of 8 symptoms was calculated. The questionnaire included sections on preventive practices and common symptoms of FBA. Participants answered yes/no questions about preventive practices and right/wrong questions about FBA symptoms. The total knowledge score was calculated by adding up scores from these sections, with a maximum possible score of 21. Afterward, a total percentage score was calculated to categorize those with bad and good knowledge.

The questionnaire was created by an expert committee consisting of 5 consultants: 2 in public health, 1 in family medicine, and 2 otolaryngologists. The committee thoroughly evaluated the content and validity of the questionnaire. The questionnaire was reviewed by experts at College of Medicine, King Khalid University for content validity. Additionally, a pilot test was conducted with 25 individuals to evaluate the questionnaire’s clarity, response rate, and completion time.

### 2.4. Defined variables

#### 2.4.1. Good/bad knowledge categories

For our study, we classified the participants based on their knowledge into 2 distinct categories: good and poor. The level of knowledge was deemed good if the score ranged between 60% and 100%, and bad if the score fell below 60%. While prior research often utilized Bloom’s cutoff point, defining 80% to 100% as good scores, 60% to 79% as moderate scores, and <60% as bad scores, our team opted for a simplified approach with 2 subdivisions instead of 3. We combined moderate and good scores into 1 category while retaining poor knowledge scores of <60%.^[25]^

#### 2.4.2. Aspiration experience

Participants who personally passed or knew someone who had passed the FBA experience were labeled as having a “positive experience.” Those who did not experience it or did not know anyone who experienced it were labeled as “negative experience.”

#### 2.4.3. Perceived seriousness

Respondents who chose FBA as the main cause of death in children under 4 years old were labeled as “serious” for perception. However, those who chose any other cause were labeled “non-serious.”

### 2.5. Statistical analysis

Statistical analyses were done by R 4.2 attached codes of analysis. Categorical variables were reported as numbers and percentages. Numerical variables were presented as mean and standard deviation. For bivariate analysis, Pearson chi-squared test was used for categorical variables, and an independent sample *t*-test was used to compare the means of quantitative variables. Cronbach’s Alpha test was used to assess the internal consistency of the study questionnaire. The first scale, focusing on practices and recommendations for preventing FBA, has a Cronbach Alpha of 0.855, indicating very good internal consistency and reliability. The second scale, which assesses knowledge about the signs and symptoms of FBA, has a Cronbach Alpha of 0.65, indicating moderate internal consistency (see Table S1, Supplemental Digital Content, http://links.lww.com/MD/N270 which illustrates the reliability Analysis of the study questionnaire). A *P* value was considered significant if it was lower than 0.05.

The data file is attached to as a supplementary file (Supplemental Digital Content, http://links.lww.com/MD/N269).

### 2.6. Ethical considerations

The study received approval from the Research Ethics Committee of King Khalid University (approval no: ECM#2023-1803). Before filling out the questionnaire, participants were provided with a clear explanation of the study objectives, procedures, and potential risks and benefits. Only those who consented to participate were included and complete the questionnaires. Participant anonymity, confidentiality (no names or IDs required), and privacy were ensured. Respondents were informed of their freedom to withdraw from the study at any time without any consequences.

## 3. Results

A total of 870 respondents participated in this study. Most of them (79.7%) were females, 48.1% were aged 18 to 30 years, 72.9% had university degrees, 30.6% were students, 26.9% worked in the educational sector, 43.6% reported monthly income of <5000 Saudi Riyals, and 19.8% of them identified themselves as health practitioners (HCPs) (Table 1).

**Table 1:** Respondents’ personal, socio-demographic characteristics.

Characteristic (N = 870)	Level	N (%)
Gender	Male	177 (20.3)
	Female	693 (79.7)
Age	18–30 years	419 (48.1)
	31–40 years	173 (19.9)
	>40 years	278 (32.0)
Education	Middle	20 (2.3)
	Secondary	171 (19.7)
	University	635 (72.9)
	Other	44 (5.1)
Occupation	Student	266 (30.6)
	Educational sector	234 (26.9)
	Health sector	75 (8.6)
	Military sector	55 (6.3)
	Private sector	50 (5.7)
	Industrial sector	19 (2.2)
	Other	171 (19.7)
Income	<5000 SAR	379 (43.6)
	5000–9000 SAR	182 (20.9)
	More than 9000 SAR	309 (35.5)
Are you a health practitioner	Yes	172 (19.8)
	No	698 (80.2)

1 USD (United Stated Dollar) = 3.75 SAR (Saudi Arabian Riyal).

**Figure 1.**
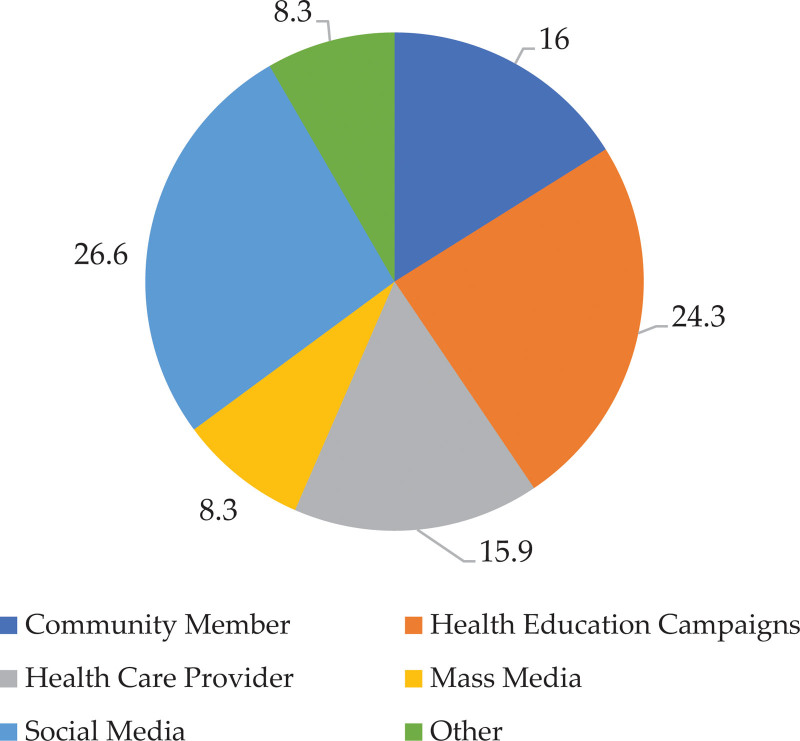
Sources of Information regarding FBA among the studies sample.

The sources of knowledge regarding FBA are illustrated in Figure 1. Mass was the least mentioned source (8.3%) while social media (26.6%) was the most cited followed by health education campaigns (24.3%).

More than half of the participants 53.3% perceived FBA as a serious health problem. A considerable sector (50.9%) of respondents reported knowing someone who had experienced FBA. Nearly three-fourths of the respondents (70.6%) reported having experienced FBA themselves and 72.0% experienced aspiration incidents beyond their first year of life. The distribution of FBA reveals a diverse range of materials, including organic (food and plant), electrochemical (e.g., battery), and inorganic (metals, minerals) substances. Back blows and chest thrusts were the most commonly employed strategies to manage FBA (32.6%), followed by the Heimlich maneuver with the help of another person (21.7%), and coughing (22.1%) (Table 2).

**Table 2:** Respondents’ experience with foreign body aspiration (FBA).

Characteristic (N = 870)	Level	N (%)
Perceived seriousness of FBA	FB is a main cause of death in children under 4 years	406 (46.7)
	FB is not a main cause of death in children under 4 years	464 (53.3)
Does someone you know ever aspirate a FB?	No	443 (49.1)
	Yes	443 (50.9)
Did you ever aspirate a FB?	Yes	614 (70.6)
	No	256 (29.4)
Your age when you aspirated the FB? (n = 614)	≤ 1 year	172 (18.0)
	More than 1 year	442 (72.0)
Nature of your aspirated FB (n = 614)	Organic (food and plant)	339 (55.2)
	Electrochemical (ex. battery)	44 (7.2)
	Inorganic (metals, minerals)	153 (29.9)
	Other	78 (12.7)
Management of your aspirated FB (n = 614)	Coughing	136 (22.1)
	Back blows and chest thrusts	200 (32.6)
	Going to the hospital to remove it	116 (18.9)
	The Heimlich maneuver by oneself	29 (4.7)
	The Heimlich maneuver with the help of another person	133 (21.7)

The analysis suggests a significant association between educational level and FBA incidents (*P* = .035). On the other hand, there was no statistically significant association between gender, age, income level, being HCPS, occupation, income, and perceived seriousness of FBA with FBA incidents (Table 3).

**Table 3:** Analysis of factors associated with foreign body aspiration.

Variable	Level	Ever you aspirated FB		*P*
		No	Yes	
Gender	Female	197 (28.4)	496 (71.6)	0.236
	Male	59 (33.3)	118 (66.7)	
Age	18–30 years	119 (28.4)	300 (71.6)	0.811
	31–40 years	52 (30.1)	121 (69.9)	
	> 40 years	85 (30.6)	193 (69.4)	
Educational level	Middle	10 (50.0)	10 (50.0)	
	Secondary	55 (32.2)	116 (67.8)	0.035
	University	184 (29.0)	451 (71.0)	
	Other	7 (15.9)	37 (84.1)	
Being a health professional?	No	212 (30.4)	486 (69.6)	0.254
	Yes	44 (25.6)	128 (74.4)	
Perceived seriousness of FBA	FB is a main cause of death	127 (31.3)	279 (68.7)	0.294
	FB is not a main cause of death	129 (27.8)	335 (72.2)	
Income	<5000 SAR	110 (29.0)	269 (71.0)	0.974
	5000–9000 SAR	54 (29.7)	128 (70.3)	
	More than 9000 SAR	92 (29.8)	217 (70.2)	
Occupation	Educational sector	69 (29.5)	165 (70.5)	
	Health sector	15 (20.0)	60 (80.0)	0.153
	Industrial sector	10 (52.6)	9 (47.4)	
	Military sector	18 (32.7)	37 (67.3)	
	Private sector	15 (30.0)	35 (70.0)	
	Student	74 (27.8)	192 (72.2)	
	Other	55 (32.2)	116 (67.8)	

1 USD (United Stated Dollar) = 3.75 SAR (Saudi Arabian Riyal).

FBA = foreign body aspiration.

Table S2, Supplemental Digital Content, http://links.lww.com/MD/N271 illustrates knowledge of FBA and preventive practices among the study sample across the 21 questions. Poor knowledge was identified among 75.3% of participants. The poor knowledge was higher among the age group 31 to 40 years at a rate of 83.3% (*P* < 0.001). This poor knowledge was also significantly higher among non-HCPs (63.4% vs 36.6%, *P* < 0.001) and people who perceive FBA as a serious incident (80.8% vs 70%, *P* < 0.001). Gender, education, and aspiration experience were not significantly associated with participants’ level of knowledge (Table 4).

**Table 4:** Participants’ knowledge regarding FBA and their socio-demographic, personal characteristics, perceptions, and experiences.

Characteristic		Good knowledge	Poor knowledge	*P*-value
		n = 215 (24.7%)	n = 655 (75.3%)	
Gender	Female	178 (25.7)	515 (74.3)	0.223
	Male	37 (20.9)	140 (79.1)	
Age	18–30 years	131 (31.3)	288 (68.7)	<.001
	31–40 years	28 (16.2)	145 (83.8)	
	> 40 years	56 (20.1)	222 (79.9)	
Educational level	Middle	5 (25.0)	15 (75.0)	0.786
	Secondary	43 (25.1)	128 (74.9)	
	University	159 (25.0)	476 (75.0)	
	Other	8 (18.2)	36 (81.8)	
Are you a health practitioner	No	152 (21.8)	546 (78.2)	<.001
	Yes	63 (36.6)	109 (63.4)	
Aspiration experience	No	102 (23.9)	325 (76.1)	0.635
	Yes	113 (25.5)	330 (74.5)	
Perceived seriousness	Serious	78 (19.2)	328 (80.8)	<.001
	Not serious	137 (29.5)	327 (70.5)	

Table 5 summarizes respondents’ knowledge regarding FBA based on their profession, aspiration experience, and perceived seriousness of FBA. The data is divided into categories showing correct and incorrect responses for managing an aspirated infant and adult, alongside the mean knowledge scores for symptoms and preventive practices of FBA. The correct knowledge on the first step to save an aspirated infant was similar across all groups, with no significant differences. However, significant differences were noted in the knowledge of the first and second steps to save an aspirated adult, with HCPs and those who perceived FBA as serious showing higher correct response rates (*P* < .001). Additionally, mean knowledge scores for symptoms and preventive practices of FBA were significantly higher among those who considered FBA serious and among HCPs (*P* < .001).

**Table 5:** Respondents’ knowledge regarding FBA according to their profession, aspiration experience, and perceived seriousness of foreign body aspiration.

Characteristic (N = 870)	Aspiration experience		Perceived seriousness		Health profession		Total sample
	Positive n (%)	Negative n (%)	Serious n (%)	Not serious n (%)	HCPs n (%)	Not HCPs n (%)	n (%)
*Knowledge of FBA management*							
*First step to save an aspirated infant*							
Correct	98 (14.4)	16 (10.4)	70 (15.1)	49 (12.1)	22 (12.8)	95 (13.8)	119 (13.7)
Incorrect	583 (85.6)	138 (89.6)	394 (84.9)	357 (87.9)	150 (87.2)	595 (86.2)	751 (86.3)
*P-value* †	0.192		0.196		0.738		
*First step to save an aspirated adult*							
Correct	293 (43.0)	53 (34.4)	198 (42.7)	157 (38.7)	92 (53.5)	260 (37.7)	355 (40.8)
Incorrect	388 (57.0)	101 (65.6)	266 (57.3)	249 (61.3)	80 (46.5)	430 (62.3)	515 (59.2)
*P-value* †	0.050		.0231		<.001*		
*Second step to save an aspirated adult*							
Correct	208 (30.5)	45 (29.2)	165 (35.6)	94 (23.2)	74 (43.0)	183 (26.5)	259 (29.8)
Incorrect	473 (69.5)	109 (70.8)	299 (64.4)	312 (76.8)	98 (57.0)	507 (73.5)	611 (70.2)
*P-value* †	0.747		<.001*		<.001*		
*Knowledge of symptoms of FBA*							
*Mean (SD*)	3.2 (1.8)	2.9 (1.8)	3.5 (1.8)	2.8 (1.8)	3.4 (1.9)	3.1 (1.8)	3.2 (1.8)
*P-value* ‡	0.085		<.001*		0.019*		
*Knowledge of FBA preventive practices*							
*Mean (SD*)	5.1 (3.2)	4.8 (3.4)	5.4 (3.2)	4.4 (3.2)	5.7 (3.2)	4.8 (3.2)	4.9 (3.2)
*P-value* ‡	0.373		<.001*		<.001*		
*Total knowledge score*							
*Mean (SD*)	9.2 (4.8)	8.5 (4.9)	9.8 (4.5)	7.9 (4.6)	10.3 (4.8)	8.7 (4.6)	8.9 (4.7)
*P-value* ‡	0.127		<.001*		<.001*		

† Chi-squared test.

‡ Independent sample *t*-test.

* Significant < .05.

## 4. Discussion

FBA is a serious condition that affects children and uncommonly adults. FBA needs to be diagnosed and treated as early as possible to prevent complications and permanent lung damage. Many accidental childhood deaths are caused by tracheobronchial FBA, which is a life-threatening emergency.^[26,27]^ The present study investigated the community’s previous FBA experience and their knowledge regarding FBA.

### 4.1. The main study findings

According to the findings, a large sector of participants recognized FBA as a severe health concern. Almost three-fourths of the respondents reported having personally experienced FB aspiration. This occurrence of FBA was more reported among males and beyond infancy. The aspirated FBs included a broad range of materials, such as organic, inorganic, and electrochemical substances. Back blows and chest thrusts emerged as the primary management strategy, followed by The Heimlich maneuver with the help of another person. Nearly half of respondents reported knowing someone who had experienced FB aspiration. Knowledge about FBA among participants was generally poor, especially among non-HCPs and individuals perceiving FBA less seriously. Social media and health education campaigns emerged as the primary sources of information about FBA, while mass media was less frequently cited.

### 4.2. Interpretation of the main findings

Experience of FBA, in the current study, nearly three-fourths of the sample had a positive personal FBA experience. Moreover, around one-half of the participants had nonpersonal FBA experience. On the other hand, a lower incidence was reported by Dongol et al.^[28]^ They reported an incidence of 0.37% among children. The current finding highlights the high frequency of this potentially serious condition among Asir residents. This is an alarming finding calling for serious preventive actions to be taken to decrease the incidence of FBA-related serious morbidity and mortality.

There was no significant increase in the incidence of FBA across sex and different age groups. On the other hand, previous studies reported a higher incidence of FBA among males.^[29–31]^ The higher incidence of FBA among male children may be attributed to their adventurous and impulsive behavior. Male children often engage in activities that involve exploration and risk-taking, which can increase the likelihood of encountering situations leading to FBA. Their natural curiosity and tendency to experiment with objects may also contribute to a higher incidence of FBA compared with female children.^[32]^ We speculate that the inclusion of adult population in this study is why we did not find a significant association between sex and FBA.

As to the nature of the AFB, organic FBs were the most cited type by more than half (51.6%) of the study respondents. This finding is in accordance with other worldwide studies reporting organic FBs of food origin as the most common aspirate.^[10,12,33,34]^ The difference between countries lies in the type of aspirated food which differs according to culture, residence, and food habits.^[13,35]^ In a previous study in the Asir region in the KSA, the most commonly inhaled FB was watermelon seed.^[36]^

The perceived seriousness of the FBA was reported by 46.7% of the sample which is a much lower figure than their positive FBA experience (70.6%). This may be because, based on their personal FBA experience, they were able to dislodge the object easily by coughing or using back blows and chest thrusts. Only 18.9% required hospital intervention to manage their condition. Indeed, underestimation of the condition could contribute to increased incidence of its serious consequences in the community if no appropriate actions are taken.

A common challenge highlighted in the literature regarding FBA management is the delayed diagnosis. Diagnosis delay leads to an increased incidence of complications, longer hospital stays, and further respiratory complications. FBA is frequently misdiagnosed as asthma, pneumonia, or laryngitis. Moreover, children under 3 years are more likely to receive a wrong or delayed diagnosis. The main reason behind delayed or missed diagnosis is unwitnessed aspiration incidents by parents, caregivers, or teachers which could mislead the clinician in the history taken to provide the correct diagnosis.^[3,35,37]^

Adults are expected to be the first to witness FBA incidents, whether at home, in daycares, or in settings where young children live, learn, or play. Assessing adults’ awareness levels regarding FBA showed low levels of knowledge regarding symptoms, preventive actions, and management of FBA. Only 13.7% of respondents identified the correct action to save an aspirated infant. The mean number of symptoms of FBA identified by the respondents was 3.2 ± 1.8 for the 8 symptoms listed. For the 10 preventive measures of FBA among young children, participants identified a mean of 4.9 ± 3.2 items. Generally, only 24.7% of the sample showed good total knowledge of FBA. Such findings indicate a lack of awareness among the adult population. Inadequate adult knowledge was reported in other studies conducted in KSA^[38,39]^ and in other regions and countries such as Jordan,^[40]^ Pakistan,^[27]^ India,^[22]^ Brazil,^[14]^ and Japan.^[41]^ On the contrary, a study conducted in Nigeria showed that there was good parental knowledge regarding FBA and 47.4% knew that they should not give groundnuts/seeds or small toys to children <3 years.^[42]^ The discrepancy in knowledge level should be interpreted in the light of different methodologies and knowledge assessment tools used, rather than as real differences in awareness levels.

People who showed good knowledge were more likely to be HCPs. Such finding reveals defective community-level educational activities regarding such serious incidents. Where people rely on their academic and professional education to deal with FBA events. Knowledge levels were not associated with gender or educational level. A similar absence of significant association was reported in a Brazilian study.^[14]^ However, another study conducted in Nigeria found a significant association between knowledge level and participants’ gender and education.^[42]^ This finding implies that educational efforts should target all community members regardless of gender or educational level.

Regarding the reported sources of information about FBA, social media was the most cited source this is similar to other studies conducted in region KSA^[39,43]^ However, another study conducted in Jeddah reported that HCPs were the top cited source.^[44]^ It is important to note that there are regional differences in how people seek information about FBA in KSA. To effectively raise awareness about the risks and prevention of FBA, educational interventions, and public health campaigns need to be tailored to the specific dynamics of each locality. This emphasizes the need for targeted efforts to address the issue.

### 4.3. Implications of the study

This study on FBA carried out in Saudi Arabia provides valuable insights into public health challenges and opportunities for intervention. Despite the participants’ perceived seriousness of FBA, there is a widespread lack of knowledge about its symptoms and preventive measures. This underlines the need for targeted educational campaigns through accessible platforms such as social media. The study also highlights the importance of improving healthcare practices and training protocols, as well as the crucial role of HCPs in disseminating accurate information and guiding preventive measures. Addressing these findings can help to develop comprehensive strategies to reduce FBA incidence and enhance public health outcomes in KSA.

### 4.4. Strengths and limitations

The present study focused on shedding light on a serious topic that has been continuously neglected, namely FBA. The study provides essential baseline data to support future interventions and educational efforts aimed at addressing FBA in KSA. By raising awareness of this issue, the incidence of FBA can be potentially reduced. However, the study has several limitations, including the non-probability nature of the sample, which may restrict the generalizability of the results to the entire population of the Asir region or the broader Saudi population. The use of non-probability sampling methods could introduce bias and may not accurately represent the diverse demographics and characteristics of the entire population. Finally, in future studies, it would be beneficial to consider incorporating a mixed-method approach, combining quantitative and qualitative methods. This approach can provide more comprehensive insights into the knowledge of community members about FBA.

## 5. Conclusions

The frequency of FBA experience is high among Asir residents in addition community awareness regarding FBA is unacceptably low. Training and educating parents have been shown to result in a decreased incidence of FBA in infants and its related serious consequences.^[23]^ The study highlights the necessity of educating caregivers of young children to mitigate instances and severe complications of FBA. Education initiatives can be disseminated through community, mass media, social media, or healthcare settings, emphasizing appropriate infant feeding practices and proper responses in cases of aspiration. Future research directions could concentrate on developing effective communication strategies across diverse demographics, assessing the effectiveness of community-based interventions, exploring cultural and linguistic influences on FBA awareness and healthcare-seeking behaviors, conducting longitudinal studies to monitor changes in awareness and FBA incidence rates, and fostering interdisciplinary collaboration to formulate comprehensive prevention methods.

## Acknowledgments

We would like to acknowledge the study participants who participated in this study. The authors extend their appreciation to the Deanship of Research and Graduates Studies at King Khalid University for funding this work through small under grant number group research RGP1/180/45.

## Author contributions

**Conceptualization:** Abdullah Alhelali, Lujain Khaled T Bukhari, Abeer Ali H. Hassan, Raghad Yahya AlQahtani, Ramy Mohamed Ghazy.

**Data curation:** Ayoub Ali Alshaikh.

**Formal analysis:** Ayoub Ali Alshaikh, Raghad Yahya AlQahtani.

**Funding acquisition:** Abduaelah Ali H. Hassan.

**Investigation:** Abdullah Alhelali.

**Methodology:** Ayoub Ali Alshaikh, Mohammed Yahya Althwabi Asiri.

**Resources:** Mohammed Yahya Althwabi Asiri.

**Supervision:** Abdulrahim Ali Hassan.

**Validation:** Abdulrahim Ali Hassan, Mohammed Yahya Althwabi Asiri.

**Visualization:** Reem Saud H. Alqahtani.

**Writing – original draft:** Lujain Khaled T. Bukhari, Reem Saud H. Alqahtani, Ramy Mohamed Ghazy.

**Writing – review & editing:** Abduaelah Ali H. Hassan, Ramy Mohamed Ghazy.

## Supplementary Material






