# Supplementary material for: Community experience and awareness regarding foreign body aspiration in Asir region, Kingdom of Saudi Arabia
Source: Medicine (Baltimore). 2024 Aug 2;103(31):e38869. doi: 10.1097/MD.0000000000038869 (PMC11296466; doi:10.1097/MD.0000000000038869)
Supplement: Supplementary file 1 [file medi-103-e38869-s001.docx]

**Supplementary tables**

The data presents an internal consistency analysis of two scales related to the prevention and recognition of foreign body aspiration (FBA) in children, evaluated using Cronbach's alpha. The first scale assesses practices and recommendations for FBA prevention and demonstrates a high internal consistency with a Cronbach's alpha of 0.855. This indicates that the items reliably measure the construct of FBA prevention practices. The items include encouraging children to sit while eating, avoiding foods with small objects, watching children while they eat, cutting foods like grapes lengthwise, and avoiding making children laugh or cry while eating. Each item contributes positively to the scale, with Cronbach's alpha values ranging from 0.835 to 0.852 if an item is deleted.

The second scale focuses on knowledge of signs and symptoms of FBA and shows a moderate internal consistency with a Cronbach's alpha of 0.65. The items in this scale include identifying choking, cough, breathing difficulty, and aphonia as symptoms of FBA. The analysis provides mean and variance values if each item is deleted, as well as corrected item-total correlations and squared multiple correlations, reflecting each item's contribution to the overall scale reliability. Supplementary table 1

Supplementary table 1: Reliability analysis

| Practices and recommendations for prevention of FBA in children (10 Questions) | | | | | |
| --- | --- | --- | --- | --- | --- |
|  | Scale Mean if Item Deleted | Scale Variance if Item Deleted | Corrected Item-Total Correlation | Squared Multiple Correlation | Cronbach's Alpha if Item Deleted |
| It is advisable to encourage children to sit while eating and to refrain from running or playing during meals. | 4.45 | 8.611 | 0.537 | 0.351 | 0.844 |
|  | 4.58 | 8.407 | 0.643 | 0.501 | 0.835 |
| It is not advisable to feed children foods containing small objects, such as popular sweets with a toy inside. | 4.63 | 8.468 | 0.645 | 0.505 | 0.835 |
|  | 4.42 | 8.619 | 0.535 | 0.306 | 0.844 |
| Watching children when they eat | 4.42 | 8.446 | 0.601 | 0.382 | 0.838 |
|  | 4.51 | 8.487 | 0.59 | 0.37 | 0.839 |
| Emphasize the need to cut soft and round foods, such as grapes and sausages, lengthwise before serving them to children. | 4.49 | 8.453 | 0.6 | 0.379 | 0.838 |
|  | 4.4 | 8.494 | 0.585 | 0.374 | 0.84 |
| It is not advisable to make young children laugh or cry while eating | 4.38 | 8.88 | 0.445 | 0.208 | 0.852 |
|  | 4.26 | 9.05 | 0.423 | 0.231 | 0.853 |
| Cronbach alpha | 0.855 |  |  |  |  |
| Knowledge about signs and symptoms of FBA (7 Questions) | | | | | |
| K3 .Choking | 2.4 | 2.808 | 0.219 | 0.101 | 0.621 |
|  | 2.71 | 2.691 | 0.219 | 0.066 | 0.627 |
| K3 cough | 2.45 | 2.547 | 0.381 | 0.194 | 0.576 |
|  | 2.71 | 2.428 | 0.401 | 0.2 | 0.568 |
| K3 Breathing difficulty | 2.93 | 2.625 | 0.358 | 0.211 | 0.583 |
|  | 3.06 | 2.874 | 0.319 | 0.226 | 0.598 |
| K3 Aphonia | 2.96 | 2.69 | 0.336 | 0.15 | 0.59 |
|  | 2.93 | 2.62 | 0.367 | 0.183 | 0.581 |
| Cronbach alpha | 0.65 |  |  |  |  |

The table presents a comprehensive survey of 870 participants' knowledge regarding foreign body aspiration (FBA) prevention and management in children, as well as the recognition of related symptoms. The results reveal both strengths and areas for improvement in public awareness. In terms of prevention practices, a majority (69.2%) understand the importance of children sitting while eating, but only slightly more than half (54.8%) recognize the risks associated with foods containing small objects. Over half (56.9%) acknowledge the need for supervision during meals, yet less than half (45.6%) are aware of the necessity to cut soft and round foods like grapes and sausages lengthwise. Furthermore, a significant portion (43.6%) are unaware of the risks posed by making children laugh or cry during meals, and just over half (53.3%) see the importance of teaching proper chewing techniques. When it comes to avoiding choking hazards for young children, only 52.6% understand the risks of giving certain hazardous foods. Awareness of the necessity for age-appropriate warnings on plastic bags (32.1%) and toys dispensed with food (36.9%) is notably low. Additionally, awareness of the importance of campaigns is split (49.9%), indicating a need for more advocacy. Regarding life-saving recommendations for FBA management, most participants (63.8%) correctly identify the initial step for infant choking as back blows and chest thrusts, but there is less certainty for adults, with 40.8% suggesting encouraging coughing as the first step and only 35.9% identifying back blows and chest thrusts as a subsequent step. Knowledge of FBA symptoms shows that most participants can identify choking (76.6%) and breathing difficulty (71.6%) as key symptoms, but fewer recognize coughing (45.6%), aphonia (44.9%), stridor (10.5%), severe pain (20.5%), and loss of consciousness (23.0%) as indicators.
