# Supplementary material for: Community experience and awareness regarding foreign body aspiration in Asir region, Kingdom of Saudi Arabia
Source: Medicine (Baltimore). 2024 Aug 2;103(31):e38869. doi: 10.1097/MD.0000000000038869 (PMC11296466; doi:10.1097/MD.0000000000038869)
Supplement: Supplementary file 3 [file medi-103-e38869-s003.docx]

**Table 2S: Knowledge of Foreign Body Aspiration Preventive Practices Among the Study Sample (21 Questions)**

| ***Knowledge category (N=870)*** | ***Level*** | ***N (%)*** |
| --- | --- | --- |
| Practices and recommendations for prevention of FBA in children (10 Questions) | | |
| K1. It is advisable to encourage children to sit while eating and to refrain from running or playing during meals. | Yes | 602 (69.2) |
|  | No | 268 (30.8) |
| K1.It is not advisable to feed children foods containing small objects, such as popular sweets with a toy inside. | Yes | 477 (54.8) |
|  | No | 393 (45.2) |
| K1. Watching children when they eat | Yes | 495 (56.9) |
|  | No | 375 (43.1) |
| K1. Emphasize the need to cut soft and round foods, such as grapes and sausages, lengthwise before serving them to children. | Yes | 397 (45.6) |
|  | No | 473 (54.4) |
| K1. It is not advisable to make young children laugh or cry while eating | Yes | 379 (43.6) |
|  | No | 491 (56.4) |
| K1. It is not advisable to teach children to chew slowly and correctly | Yes | 464 (53.3) |
|  | No | 406 (46.7) |
| K1. It is not advisable to give children under 4-5 years of age foods that could pose a choking hazard, such as nuts and dried snacks like popcorn, almonds, walnuts, sunflower seeds, and corn | Yes | 458 (52.6) |
|  | No | 412 (47.4) |
| K1. It is mandatory to print appropriate and age-appropriate warnings on plastic bags for toys to prevent suffocation | Yes | 279 (32.1) |
|  |  |  |
|  | No | 591 (67.9) |
| K1. Toys dispensed or mixed with food must carry the following warning: “Contains toy.” Adult supervision recommended | Yes | 321 (36.9) |
|  | No | 549 (63.1) |
| K1. Carrying out awareness campaigns about risk and dealing with inhalation of foreign bodies | Yes | 434 (49.9) |
|  | No | 436 (50.1) |
| Priority life-saving recommendations for the management of FBA in infants and adults (3 Questions) | | |
| K2. The first step is infant Management | Back blows and chest thrust | 555 (63.8%) |
|  | Encourage him to cough | 62 (7.1%) |
|  | The Heimlich maneuver | 39 (4.5%) |
| K2. Adult 1^st^ step of FB Management | Back blows and chest thrust | 301 (34.6%) |
|  | encourage him to cough | 355 (40.8%) |
|  | Heimlich maneuver | 154 (17.7%) |
|  | take it out by hand | 60 (6.9%) |
| K2. Adult 2^nd^ step of FBA management | Back blows and chest thrust | 312 (35.9%) |
|  | encourage him to cough | 214 (24.6%) |
|  | Heimlich maneuver | 259 (29.8%) |
|  | take it out by hand | 85 (9.8%) |
| Knowledge about signs and symptoms of FBA (7 Questions) | | |
| K3 Choking | No | 204 (23.4%) |
|  | Yes | 666 (76.6%) |
| K3 cough | No | 473 (54.4%) |
|  | Yes | 397 (45.6%) |
| K3 Breathing difficulty | No | 247 (28.4%) |
|  | Yes | 623 (71.6%) |
| K3 Aphonia | No | 479 (55.1%) |
|  | Yes | 391 (44.9%) |
| K3 Stridor | No | 779 (89.5%) |
|  | Yes | 91 (10.5%) |
| K3 Severe pain | No | 692 (79.5%) |
|  | Yes | 178 (20.5%) |
| K3 loss of consciousness | No | 670 (77.0%) |
|  | Yes | 200 (23.0%) |
| K3 Choking | No | 204 (23.4%) |
|  | Yes | 666 (76.6%) |
| K3 Cough | No | 473 (54.4%) |
|  | Yes | 397 (45.6%) |
| K3 Breathing difficulty | No | 247 (28.4%) |
|  | Yes | 623 (71.6%) |
| K3 Aphonia | No | 479 (55.1%) |
|  | Yes | 391 (44.9%) |
